# A randomized controlled trial to examine the impacts of disclosing personalized depression risk information on the outcomes of individuals who are at high risk of developing major depression: a research protocol

**DOI:** 10.1186/s12888-019-2270-9

**Published:** 2019-09-18

**Authors:** JianLi Wang, Glenda MacQueen, Scott Patten, Douglas Manuel, Bonnie Lashewicz, Norbert Schmitz

**Affiliations:** 1 University of Ottawa Institute of Mental Health Research, University of Ottawa, Room 5404, 1145 Carling Ave, Ottawa, Ontario K1Z 7K4 Canada; 2 Department of Psychiatry, Cumming School of Medicine, University of Calgary, Calgary, Alberta Canada; 3 Department of Community Health Sciences, Cumming School of School of Medicine, University of Calgary, Calgary, Alberta Canada; 4 Ottawa Hospital Research Institute, University of Ottawa, Ottawa, Ontario Canada; 5 Douglas Mental Health Institute, Department of Psychiatry, McGill University, Montreal, Quebec Canada

**Keywords:** Major depression, Multivariable risk predictive algorithms, High risk, Early identification, General population, Self-help, Psychological distress

## Abstract

**Background:**

Major depressive disorder is one of the most prevalent and disabling forms of mental illness in the general population. One public health strategy that may reduce the disease burden is early identification and prevention - identifying people who are at high risk and intervening to prevent symptoms from progressing into a major depressive episode (MDE). Multivariable risk predictive algorithms (MVRP) have been developed to estimate personalized risk (probability) of an MDE. The purpose of this trial is to answer the questions: (1) Does disclosure of personalized depression risk information promote high-risk individuals to take preventive actions? (2) Will disclosure of personalized depression risk information negatively affect the mental health of those at high risk?

**Methods:**

We are recruiting 350 high-risk men and 350 high-risk women across the country. Individuals are eligible, if they: (1) are 18 years or older, (2) have not had a depressive episode in the past two months, (3) are at high risk of MDE based on the MVRPs (predicted risk of 6.5% + for men and of 11.2% + for women), (4) can communicate in either English or French, and (5) agree to be contacted for follow-up interviews. The MVRPs were developed and validated using longitudinal data from over 10,000 Canadians across the country. Eligible participants are randomized into (1) the control group, and (2) the group receiving personalized depression risk information. The participants are assessed at baseline, 6 and 12 months regarding accuracy of risk perception, use of self-help strategies and changes in psychological distress and functioning. Qualitative interviews are conducted in sub-samples of the intervention groups to explore how the personalized information affects risk perception, self-help behaviors and mental health.

**Discussion:**

MVRPs can be used for risk stratification and planning preventive actions. The personalized risk information produced by MVRPs may also empower users to actively engage in self-management. This trial will contribute to the knowledge base about the potential health benefits and psychological harms associated with the provision of personalized depression risk information that will inform future implementation and patient-physician communication in the clinical settings.

**Trial registration:**

NCT02943876. Date of trial registration: October 21st, 2016.

## Background

There is a pressing need for prevention of major depressive disorder (MDD). The Global Burden of Disease study [1] reported that MDD was the #2 leading cause of disease burden worldwide in 2010. Despite a significant increase in mental health service use in the past two decades, there has been no a measurable change in the prevalence of MDD in various countries [2]. The unchanged disease burden associated with MDD suggests that more effective efforts in early identification and prevention are needed, e.g., identifying people who are at high risk and taking preventive actions to lower the risk.

In medicine, multivariable risk prediction (MVRP) models are often used to estimate an individual’s absolute risk (probability) of developing a disease in a given time period, based on the individual’s current exposure to a key set of known risk factors (i.e., baseline risk). Well-known examples include the Framingham risk prediction algorithms for cardiovascular disease [3]. The Framingham risk algorithms are used by clinicians in predicting the risk of developing coronary disease in individuals free of the disease. The Framingham risk functions underpin several of the current policies for preventive interventions, including statin therapy for those with relatively high risk of cardiovascular disease.

There is a paucity of research in risk prediction for mental disorders. This is partly due to the lack of population-based longitudinal studies on mental disorders with frequent assessments. In 2013, our team developed and validated sex-specific MVRPs for MDE in the Canadian general population [4]. The MVRPs were developed to predict 4-year risk of MDE, using longitudinal data from 4737 men and 5864 women who were randomly selected across Canada, and who had not had a MDE in the past year prior to the baseline. The MVRPs includes questions about personal and family history of MDD, ongoing negative life stressors and childhood traumatic experience. Predictors in the MVRPs are in Table 1. The MVRPs had good discriminative power (men: C = 0.7953; women: C = 0.7667), and excellent calibration with the data. In men, the observed and predicted 4-year risk of a MDE was 5.15% and 5.25%, respectively; in women, the observed and predicted 4-year risk of a MDE was 8.27 and 8.31% [4]. We validated the MVRPs in Canadians followed during a different time period [4].

**Table 1:** The predictors in the sex-specific multivariable risk predictive algorithms for major depression

	Prediction Algorithm for Women	Prediction Algorithm for Men
Predictors that do not change with time	1. Age (continuous)	1. Age (continuous)
	2. Past major depressive episode (MDE)	2. Past major depressive episode
	3. Family history of MDE	3. Family history of MDE
		4. Physician diagnosed diabetes
	Childhood Trauma	Childhood Trauma
	4. Being traumatized for years	5. Being sent away from home
	5. Being sent away from home	6. Parents divorced
		7. Parents abused alcohol/drugs
Predictors that change with time	6. Annual personal income	8. Self-rated chronic stress
	7. Self-rated health	9. Others expect you too much
	8. Activity restrictions	10. Lack of money
	9. Satisfaction with oneself	11. Feeling everything is an effort
	10. Self-rated chronic stress	12. Took anti-depressants last month^a^
	11. Others expect you too much	13. Took sleeping pills last month^a^
	12. Family member in bad health	14. Being physically attacked in the past year
	13. Daily smoking	15. Partner had unwanted pregnancy in the past year
	14. Changed job for a worse one in the past year	
	15. Major financial crisis in the past year	
	16. Having depressed mood/lost of interest for 2 weeks in the past year	
	17. Talked to health professionals for mental health issues in the past year. ^a^	

^a^Service and medication use indicate severity of stress, rather than increased risk

MVRP tools may not only enable health professionals to identify high risk people, but also serve as communication tools to inform consumers about their health status and to empower them to actively engage in self-management. One goal of personalized risk estimates is to promote involvement of consumers in health decisions [5]. Research in cardiology and oncology [5, 6] shows that disclosing personalized risk to consumers is an effective method to achieve consumer involvement in health decisions. Because risk prediction models for MDD are new and the literature on risk disclosure is absent in psychiatry, we have no knowledge about whether provision of this information improves risk perceptions and whether high risk people will act upon the information to engage in self-help. Second, since 2013, we have directly engaged over 500 policy makers, clinicians and the general public to disseminate the prediction tools. Stakeholders have consistently indicated that a question needs to be answered before implementation: *will the provision of personalized depression risk information lead to increased psychological distress in high risk people*? As risk prediction models are new in psychiatry, there are no studies that address these notable knowledge gaps. Clearly, we need to provide answers to these practice and policy pertinent questions before moving forward to implementation.

## Methods/design

Given the background, the aim of the proposed randomized controlled trial is to answer the following research questions: (1) Does disclosure of personalized depression risk information promote high-risk individuals to take preventive actions? The effect of risk prediction may be maximized if these individuals actively engage in early prevention. (2) Will disclosure of personalized depression risk information negatively affect high-risk people’s mental health status? To safely implement the prediction algorithms, we need to ensure that the disclosure will not lead to increased psychological distress.

This study is a mixed-methods randomized controlled trial (RCT) with an embedded qualitative component. The RCT has one intervention arm (receiving personalized depression risk information) and one control arm (1:1). The target population are individuals in the community who are at high risk of major depression. The personalized depression risk is generated using the sex-specific MVRPs for MDE that we developed in Canadians aged 18+ years old [4]. Thus, the inclusion criteria are:

- no MDE at baseline, or if had a MDE in the past 12 months, the individuals were in full remission for at least 2 months before the interview (see below the question),
- aged 18+ years,
- at high risk of MDE based on the algorithms (predicted risk of 6.5% + for men and of 11.2% + for women) [4],
- agreement to be contacted for follow-up assessments, and
- no language barriers to English or French.

The status of remission was assessed by the question: “In the past 2 months or longer, has your mood been much improved or back to normal AND you DIDN’T have the symptoms of.....?” This question was adopted from the US National Epidemiological Survey on Alcohol and Related Conditions [7]. Because the prediction algorithms are sex-specific, we are recruiting 350 men and 350 women at baseline. After baseline assessment for eligibility, participants are randomized into intervention and control groups, in men and women separately.

**Fig. 1:**
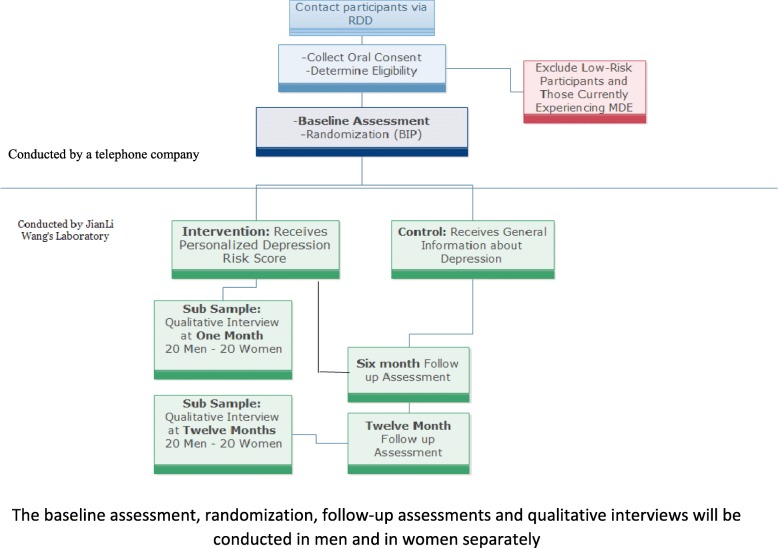
Project operational flowchart

Based on systematic reviews on risk communication [5, 6], the trials targeting behavioral and health status changes required 6 months to 12 months follow-up. Therefore we are following participants for 1 year with follow-up assessments at 6 and 12 months. The RCT adheres to CONSORT and SPIRIT guidelines [8]. An operational flow chart is in Fig. 1. The baseline and follow-up data collection was carried out using Computer Assisted Telephone Interview which automatically saves the data once the interview is completed. To obtain in-depth information about how the personalized depression risk information is processed by participants and how the information affects them emotionally, we conduct qualitative interviews 1 month after the personalized risk information is disclosed. To understand how the personalized risk information affects participants’ health behaviors, we conduct another round of qualitative interviews at 12 months. All collected data will be kept in a pass-word protected computer in the principle investigator’s office which is under 24/7 security surveillance. Only the project coordinator who is not involved in randomization and data collection has access to the data with personal identification information. Data without personal identification information will be analysed. Only aggregate results will be presented and published. This study has been approved by the Ethics Review Board of the Royal Hospital, Ottawa, Canada, and is reviewed by ERB on an annual basis.

### Recruitment

The target population for future preventive studies is high risk people in the general population. For the proposed study, we are recruiting eligible participants using the random digit dialing method (RDD). We have used the RDD for recruitment in other longitudinal studies [9, 10] and an ongoing national RCT [11]. Recruitment, screening, baseline assessment and randomization are completed by a telephone survey firm that has access to household telephone and validated cell phone numbers across the country. The recruitment and randomization procedures and questionnaire were pilot tested in 20 eligible participants, using a cognitive interviewing method [12].

A random sample of landline and cell phone numbers are selected. When a household is reached, the person who is 18+ years is assessed for eligibility. If a household has 2+ persons aged 18+ years, one is randomly selected. The interviewers explain the study objectives and procedures and answer questions. Potential participants are ensured confidentiality, that participation is voluntary and that they may withdraw at any time. Oral consent is obtained before assessment of eligibility by asking the question: “Do I have your consent to begin the survey?” The answer of “yes” and continued participation is deemed to be informed consent. The Research Ethics Board formally approved this consent.

Outcome measures are assessed at baseline, 6 and 12-month.

#### Perception of depression risk is assessed by asking

“How likely are you to get depression in the next 4 years?“ [13, 14] The answer can range from 0 to 100, where 0 = certain not to happen and 100 = certain to happen [13, 14].

*Self-management strategy use scale (SSUS)* was developed and validated by Morgan and Jorm [15]. The.

SSUS assesses the frequency of using each of the 14 self-help strategies [15], including strategies supported by research evidence (e.g., physical exercise,20–23 mindfulness relaxation,24;25 and online cognitive behavior therapy [16, 17]). Frequency of use can be rated on a 5-category scale. The SSUS has good internal consistency (Cronbach’s a = 0.80) [15].

*The Non-Specific Psychological Distress (K10)* is a 10-item screening scale intended to yield a global measure of distress based on questions about anxiety and depressive symptoms that a person has experienced in the most recent 4 week period [18]. The scale strongly discriminated between community cases and non-cases of DSM-IV disorders, with areas under the Receiver Operating Characteristic curve of 0.87–0.88 for disorders having Global Assessment of Functioning (GAF) scores of 0–70 and 0.95–0.96 for disorders having GAF scores of 0–50.28 We will compare the changes in the K10 scores over time between the groups to assess whether disclosing the personal risk leads to more psychological distress.

*Functioning impairments* is assessed by the question asking how the symptoms in the K10 affect functioning at home, work and school. We will also ask participants their number of days off work due to health problems in the past month.

*Sex-specific MVRPs for MDE* are administered to identify individuals who are at high risk for MDE, determine eligibility and assess accuracy of *perception of depression risk* over time. The algorithms have good discrimination (C statistic of 0.76 for women and of 0.79 for men), which is consistent with the range of C statistics of risk algorithms (0.75–0.80) in cardiology [19].

*Other baseline measures* include the *Composite International Diagnostic Interview – Short Form for Major Depression* (CIDI-SFMD) is administered to determine eligibility for participation. The CIDI-SFMD is a structured diagnostic interview for MDE in the past year and has been used in all cycles of the National Population Health Survey conducted by Statistics Canada, based on the DSM-IV criteria [20]. The CIDI-SFMD was developed and validated at the University of Michigan [21]. Additionally we collect data about *demographic* and *socioeconomic* characteristics and *mental health service use* (at baseline and follow-up) using standard questions from Statistics Canada surveys.

### Baseline assessment and randomization

#### Screening

Once a potentially eligible participant is identified, the interviewer from the telephone survey firm confirms the participant’s age and administers the CIDI-SFMD and the sex-specific prediction algorithms. Interviewees who are in a MDE or are below the risk thresholds based on the risk calculators, are excluded. Individuals with MDE are encouraged to contact family doctors and information about local mental health resources is provided. For those who are at low risk, the web site of the MVRPs is provided so they may monitor their risk in the future.

#### Baseline assessment

In eligible participants, the interviewer administers the K10, SSUS, and asks questions about absenteeism and perceived risk of MDE. Baseline assessment takes 20 to 25 min.

*Randomization* is carried out in men and in women. The telephone survey firm uses a survey software tool built by Voxco. The tool contains a random number generator which randomly creates a digit when the telephone script reads the function. The firm confirmed that this is comparable to the traditional method of using sealed opaque envelopes.

### Intervention and control

For the participants in the intervention group, the personalized risk is disclosed and the interviewer informs the participants that they will be contacted again at 6 months and 12 months. The interest in receiving such personalized depression information has been confirmed by our recent pilot study using the same sampling method. Our pilot data (*n* = 200) showed that 100% of high-risk individuals were interested in knowing their risks. Participants in the intervention group are also informed that some may be contacted in 1 month for a 30-min qualitative interview. A package including the following materials is mailed to intervention participants: (1) thank-you letter, (2) general information about MDE, (3) self-help strategies [15] and a summary of research evidence supporting the effectiveness of self-help strategies, and (4) $20 incentive as appreciation of their participation. For participants in the control group, the interviewer informs them that they will be contacted again at 6 and 12 months. Their personal risks will be provided at the 12-month interview. They receive the same package as those in the intervention group.

### Blinding and follow-up assessments

The telephone survey firm securely transfers encrypted baseline data to the PI on a weekly basis. The group assignment data are transferred in a separate file. The follow-up assessments are conducted at the telephone interview laboratory at the University of Ottawa Institute of Mental Health:

- One month before the scheduled follow-up interviews, letters are sent to participants to remind them of the upcoming interview.
- After the 12-month interview, participants’ group status is linked with interview data by study ID numbers.

Investigators are blinded to participants’ group status. The interviewers who conduct randomization, are not involved in follow-up interviews. The interviewers who conduct the follow-up interviews in Ottawa do not have access to participants’ group status. Given our description of study objectives, participants may know their group status. Therefore, it is possible that some participants in the control group may try to find more information about personalized depression risk. At the follow-up assessments, we will ask if they have used any risk prediction tools over the study period. At the follow-up assessments, if participants develop a MDE, they are encouraged to contact family doctors and information about local mental health resources is provided.

### Qualitative interviews

To obtain in-depth information about how disclosing personalized depression risk affects participants’ decision processes, mental health and health behaviors, we conduct two rounds of qualitative interviews via telephone, 1 month after these participants receive the personalized depression risk and at 12 months. Each includes an initial random sub-sample of 20 men and 20 women from the intervention groups. The qualitative interviews strengthen our study as we will use the findings to “triangulate” our quantitative results and to guide interpretation of the quantitative results [22]. The interviews are audio recorded. Qualitative interviews are transcribed verbatim then analyzed inductively for themes. Our analysis follows the interpretive practices of constant comparison and attempt to uncover patterns both within and between interviews [23]. Nvivo 10 software is used to support thematic analysis. We expect to achieve theoretical saturation with the initial sample of 20. However, if new themes continue to emerge in our final interviews, we will interview additional participants until no new themes emerge.

### Data monitoring

The principal investigator (PI) and the research coordinator (RC) are responsible for daily operation of the project, and monitoring data collection, data quality and potential adverse events. The PI and RC report to the research team at teleconferences held every 3 months. The funder plays no role in the process of data monitoring.

### Adverse events/harm

Our study population are individuals who are not in an episode of depression, but are at high risk. The data collection is conducted via telephone. Therefore, the possibility of physical injuries is minimum. In the circumstance that the participant may need mental health services, the interviewers are instructed to encourage the individual to seek professional help, and provide information about local mental health resources. If an unintended adverse event occurs, the ERB will be immediately notified and the event will be jointly reviewed by the board and the research team.

### Statistical analysis

All analyses will be carried out in men and in women separately, and by group assignment. We will perform an intention-to-treat analysis based on randomization. Each outcome will be analyzed with a separate regression model that includes intervention assignment and demographics, history of MDE and predicted risk at baseline as covariates.

Mixed ANOVA with a random intercept will be used to examine the effect of disclosing personalized depression risk information on changes in the SSUS scores, K10 scores and number of days off work. The mixed model will enable the repeated measures to be included in a single analysis and so that data from subjects not followed for the full year can be included. We expect no significant differences between the groups in changes of K10 scores and absenteeism at 6 and 12-month, i.e., disclosing the risk information does not lead to psychological and functioning harms. To examine the effect of risk disclosure on accuracy of perceived risk, we will first subtract participants’ perceived risk from the predicted risk. Positive values of the difference indicate underestimation of risk; negative scores indicate overestimation. We will recode difference scores into a dichotomous variable (≤10% vs > 10%) [13, 14], indicating whether perceived risk is “close” to the predicted risk. The proportions of accurate risk perception at baseline 6- and 12-month will be estimated and compared. Stratified analyses by demographic variables, history of MDE, baseline predicted risk levels will be conducted. Additionally, we will conduct the same analyses in participants who do not have missing outcome data (the completers).

Interim analysis will be conducted after 6-month follow-up. If the intervention group has a significantly higher incident proportion of major depression, and/or of suicidal behaviors, controlling for baseline covariates, than the control group, a team meeting involving staff of ethics review board will be held to review the results and determine whether the trial will be terminated.

### Sample size calculation

A RCT on the impact of e-mail promotion of self-help strategies for depression [15] showed that participants in the intervention group had modest but significant improvement in SSUS scores than those in the control group by a mean of 2.6 points, effect size d = 0.40. Assuming our study will achieve similar effect size, 258 participants (129 in each group) are needed to achieve the power of 0.80 at the α level of 0.05. The sample size calculation was done using STATA version 13. Assuming that the 12-month follow-up response rate is 75% with $20 incentive [10], we should recruit at least 344 participants (172 in each group) at baseline. Because the study will be carried out by sex separately, we proposed to recruit 350 men and 350 women who meet the inclusion criteria at baseline.

## Discussion

### Current status

At the time of submission of this manuscript, we just completed baseline recruitment of 350 men and 350 women who meet the inclusion criteria. It is anticipated that the 6-month follow-up interviews will be completed by the end of September, 2019; 12-month follow-up interviews will be completed by the end of March, 2020.

### Potential risk and mitigation strategies

We acknowledge concerns about the changes in response rates in telephone surveys due to cell phone use and telemarketing. Including eligible participants across the country will enhance the generalizability of the study. Given the vast geographic area of Canada, RDD is the only feasible method. The goal of this study is to recruit participants for a RCT, rather than selecting a representative sample. In a RCT, selection bias is not a serious concern as long as the bias is the same across the intervention and control groups [24]. To mitigate the risk, the telephone survey firm will also access the validated cellphone database. However the use of cellphone numbers is associated with increased costs. Another potential risk of the proposed study is attrition which may incur selection bias. The population-based cohort studies on mental disorders in the workplace, conducted in our lab, showed that we could achieve 77% response rate at 1 year follow-up without any financial incentives [10]. Our strategies for reducing attrition will include appropriately designed introductory scripts, a minimum of nine call back attempts spaced over weekdays and times of day and provision of a $25 incentive for each completed interview.

Finally, those deemed low risk, who develops a MDE will be excluded from the RCT at the screening stage, which is a limitation. We have planned to provide the risk prediction algorithms so that they can monitor their risks in the future.

*Knowledge translation* will be in the form of peer-reviewed publications, conference presentations and research website dissemination. We will inform the stakeholders (decision makers and health professionals) who raised the pertinent questions in our previous KT activities, about the benefits of risk disclosure through the Canadian Depression Research Intervention Network and national professional organizations with which we are closely connected. The authorship of the publications generated from this trial will be determined according to the BMC Medical Research Methodology authorship guidelines. No scientific writers will be used.

A key step in early identification and prevention of MDE is the development and implementation of advanced tools for identifying individuals who are at high risk. Our team has developed the sex-specific MVRPs for MDE. The proposed trial will develop an evidence base for guiding the disclosure of personalized risk information and understanding the process of risk communication and consumer empowerment, contributing to the advancement of early prevention of MDE in Canada and beyond.

## Data Availability

Not applicable.

## Abbreviations

CIDI-SF: Composite International Diagnostic Interview – Short Form
DSM-IV: Diagnostic and Statistical Manual for Mental Disorders – 4th edition
ERB: Ethics review board
GAF: Global Assessment of Functioning
K10: Non-specific Psychological Distress Scale
MDD: Major depressive disorder
MDE: Major depressive episode
MVRP: Multivariable risk predictive algorithms
PI: Principal investigator
RC: Research coordinator
RCT: Randomized controlled trial
RDD: Random digit dialing
SSUS: Self-management strategy use scale
